# Linac-Based Radiosurgery Treatment for a Pineal Parenchymal Tumor

**DOI:** 10.7759/cureus.96789

**Published:** 2025-11-13

**Authors:** Hali Morrison, M. Salman Faruqi, Kundan Thind, Nicolas Ploquin

**Affiliations:** 1 Department of Medical Physics, Arthur J.E. Child Comprehensive Cancer Centre, Calgary, CAN; 2 Department of Oncology, University of Calgary, Calgary, CAN; 3 Department of Radiation Oncology, Arthur J.E. Child Comprehensive Cancer Centre, Calgary, CAN; 4 Department of Radiation Oncology, Henry Ford Health, Detroit, USA; 5 Department of Medicine, Michigan State University, East Lansing, USA

**Keywords:** case report, linac-based srs, pineal parenchymal tumor, stereotactic radiosurgery, vmat

## Abstract

Pineal parenchymal tumors (PPTs) are uncommon in general and rare in the adult population. Currently, the optimal treatment for PPT of intermediate differentiation (PPTID) in older patients is unknown. Stereotactic radiosurgery (SRS) has been used as both primary and adjuvant therapy, with single or fractionated doses using Gamma Knife (Elekta, Stockholm, Sweden) or CyberKnife (Accuray Inc., Madison, WI). This article presents the case of a 77-year-old woman with a biopsy-confirmed PPTID. She was treated with single fraction VMAT (volumetric modulated arc therapy)-based SRS on a Varian TrueBeam Edge linac (Varian Medical Systems, Palo Alto, CA) using multiple non-coplanar arcs. Contouring and treatment planning were performed on contrast-enhanced MRI and CT images. Accurate patient set-up and immobilization were achieved with an open-faced thermoplastic mask, real-time motion management using an optical surface monitoring system, and kV CBCT prior to each arc. The patient has shown excellent response for tumor size with frequent follow-up up to 84 months, but 25 months later developed double-vision and headaches with MRI revealing decreasing tumor size but enhancement and FLAIR changes in the adjacent brain parenchyma suggestive of radiation necrosis. These changes stabilized as of 32 months of follow-up, and then started to decrease by 35 months.

## Introduction

Tumors involving the pineal gland are rare in adults, accounting for <1% of all central nervous system (CNS) tumors [1]. Given the location of the pineal region, patients frequently present with hydrocephalus and/or visual symptoms [2,3]. It is important to determine the histology of tumors in this location, as the management and outcomes of primary tumors of the pineal gland vary by subtype. Standard staging at presentation includes an MRI of the neuroaxis and cerebrospinal fluid (CSF) sampling either at the time of surgery or before. An endoscopic third ventriculostomy and biopsy is frequently performed to both obtain histology and relieve hydrocephalus if present.

Pineal parenchymal tumors (PPTs) account for 15-30% of pineal region tumors and are further subdivided into one of three histological classifications: pineocytoma, pineoblastoma, and PPTs of intermediate differentiation (PPTID) [1,4]. Optimal treatment for a PPTID in older patients is highly variable owing to their natural course, which can lead to leptomeningeal disease in a lower percentage of patients than pineoblastoma. A literature review revealed that for PPTID, about a third of patients received local radiation only, whereas another third received craniospinal irradiation (CSI), with 23% of patients receiving chemotherapy, demonstrating the wide variety of standard practice for this subtype [5].

Inclusion of radiotherapy for PPT treatment has most commonly been delivered by means of stereotactic radiosurgery (SRS), either with Gamma Knife (Elekta, Stockholm, Sweden) or CyberKnife (Accuray Inc., Madison, WI), although there is much variability among SRS protocols used for these patients with doses ranging from 12 to 20 Gy for a single-fraction treatment [6-12]. This article presents the case of a 77-year-old female diagnosed with a PPTID who was treated with single fraction VMAT (volumetric modulated arc therapy)-based SRS using a Varian TrueBeam Edge linear accelerator (Varian Medical Systems, Palo Alto, CA). This case examines the outcomes and risks associated with treating a target volume adjacent to the brainstem using a frameless approach (and thus requiring a standard 1 mm treatment margin) with long-term follow-up. Risk comparison with a typical frame-based (0 mm treatment margin) approach is also performed.

## Case presentation

Clinical case

**Figure 1:**
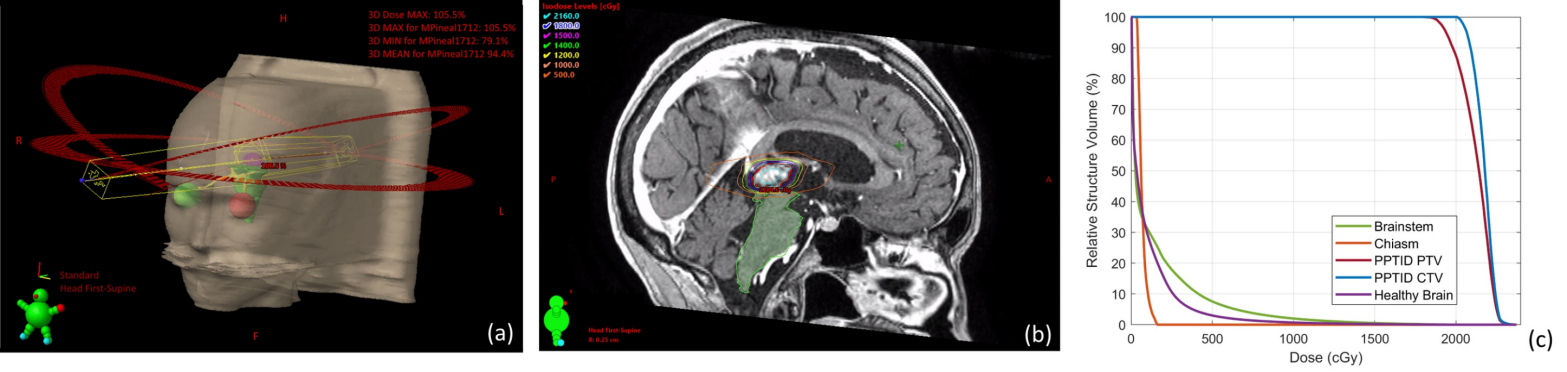
Patient treatment plan with three non-coplanar low couch angle arcs (a); dose distribution overlaid on the magnetic resonance imaging (MRI) with the clinical target volume (CTV) contour in red and the brainstem in green (b); dose-volume histogram (DVH) of relevant structures (c).

**Figure 2:**
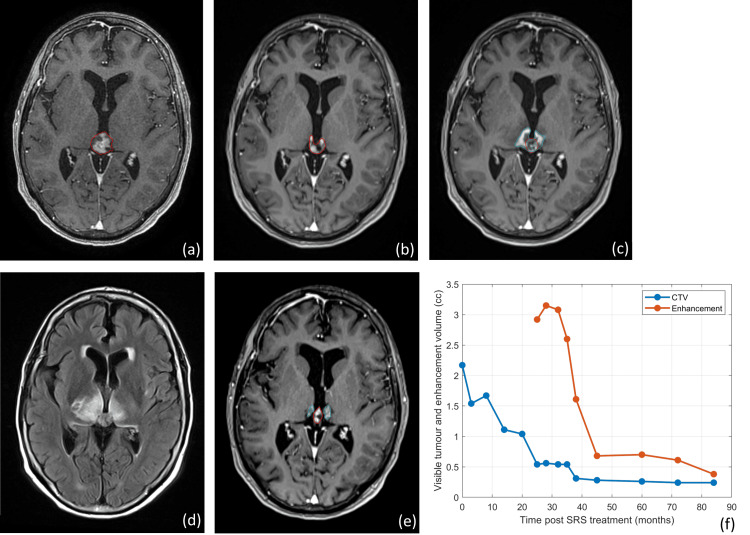
T1 post-gadolinium magnetic resonance imagings (MRIs) at the time of treatment (a), 20 months post-treatment (b), 25 months post-treatment (c), 25 months post-treatment flair MRI (d), and 84 months post-treatment T1 (e). The pineal parenchymal tumor of intermediate differentiation (PPTID) clinical target volume (CTV) as detected at the given time point are shown on each T1 image in red, as well as the enhancement region detected starting at 25 months post-treatment in blue. The CTV volume and enhancement region volume at each time point since it developed are plotted in panel (f).

A 77-year-old female presented to the emergency room (ER) with a nine-month history of incontinence and a three-month history of gait instability, headache, and cognitive decline. MRI at the time of the ER visit revealed a pineal mass with low signal intensity on T1- and high signal intensity on T2-weighted images, heterogeneous enhancement, and was causing hydrocephalus (see Figures 1, 2). Three months following the MRI, she underwent an endoscopic third ventriculostomy, tumor biopsy, and insertion of an extraventricular drain. The final pathology revealed a PPTID. CSF showed no malignant cells at both the time of surgery and four weeks post-operatively. MRI imaging showed no evidence of leptomeningeal disease. Her medical history, previous to the symptoms attributed to the lesion, included glaucoma and migraine headaches. After discussion with the patient regarding her treatment options, with consideration of the logistics of conventional fractionated versus single-fraction SRS treatment, it was decided to proceed with VMAT-based SRS with a TrueBeam Edge linac and mask immobilization system. A balanced approach was taken, given the patient's age, tolerability vs. aggressiveness of treatment, literature evidence to support single-fraction treatment, as well as practicality for the patient, considering that she lived approximately three hours' drive away from the treatment center. At the time of radiosurgery, her tumor measured 16.6 x 15.6 x 13.7 mm on T1 post-gad MRI with a volume of 2.17 cc. Radiation treatment occurred three months post-biopsy.

Treatment method

High-resolution pre-treatment MRI was performed two days before treatment. The post-gadolinium contrast-enhanced FSPGR T1 image was used to contour the clinical target volume (CTV), which was found to measure 2.17 cc. The contouring of the tumour volume was performed by the treating radiation oncologist (RO) and verified by a second RO. Particular care was taken to identify the interface between the brainstem and the PPT, as it was located immediately superior to the brainstem.

One day prior to treatment, a high-resolution contrast-enhanced CT scan was obtained, at which time a CDR open-faced mask (CDR Systems, Inc., Calgary, Canada) was created and included in the CT image. The CT image was fused with the MRI image using rigid image registration. A planning target volume (PTV) margin of 1 mm was applied, resulting in a 3.4 cc PTV volume.

The treatment plan was created using Eclipse for a Varian TrueBeam Edge linac with HDMLCs. The plan consisted of three non-coplanar arcs with couch and collimator angles optimized to avoid the brainstem as much as possible. Beam trajectories are shown in Figure 1A.

The minimum peripheral dose (prescription dose) was 18.0 Gy prescribed to the 80% isodose line. The delivery energy was 10FFF, allowing a maximum dose rate of 2400 MU/min. A summary of dosimetric properties for the PTV, brainstem, healthy brain (brain minus CTV minus CSF), and whole brain (including CTV and CSF) is summarized in Table 1. The dose distribution is shown in Figure 1B. The maximum dose delivered to any of the optic structures was 1.6 Gy (optic chiasm), well below tolerance doses [13,14]; therefore, no adverse visual effects are expected. A dose-volume histogram (DVH) plot of the relevant structures is given in Figure 1C.

**Table 1:** Summary of dosimetric and physical parameters *CI is defined as the ratio of the prescription isodose volume to the PTV volume. GI is defined as the ratio of the 50% isodose volume to the 100% isodose volume (when prescribing to the 80% isodose, the 50% in this definition is the 40% isodose).

Structure	Dosimetric or physical parameter	Value
CTV	Volume	2.17 cc
PTV	Volume	3.4 cc
	Prescription dose	18.0 Gy
	D_max_	23.7 Gy
	D_mean_	21.2 Gy
	D_min_	17.8 Gy
	V_18Gy_	99.99%
	CI*	1.21
	GI*	3.93
Brainstem	V_10Gy_	0.45 cc
	V_12Gy_	0.19 cc
	V_15Gy_	0.08 cc
	D­_max_ (0.03cc)	18.4 Gy
Healthy brain (brain minus CTV minus CSF)	V_10Gy_	10.2 cc
	V_12Gy_	6.5 cc
	V_14Gy_	4.2 cc
Whole brain (including CTV and CSF)	V_12Gy_	9.4 cc

Routine plan QA was performed using EPID analysis and delivering each treatment beam to the Octavius SRS detector (PTW Freiburg GmbH, Freiburg, Germany), using gamma criteria of 3%/1mm. Pass rates were ≥98.5% for all beams for the EPID measurements and ≥99.1% for the Octavius measurements. Winston-Lutz QA was performed daily on the treatment unit prior to any SRS patient treatments, with mean deviation required to be <0.5 mm and maximum deviations <0.75 mm.

Initial patient set-up was performed using the in-room lasers, followed by on-board imaging kV cone-beam CTs (CBCTs). At least one CBCT was performed prior to each arc being delivered to correct for any intra-fraction motion. Additional CBCTs were acquired until the maximum required shift in any direction was 0.06 cm or 0.3° in 6 DoF (degrees of freedom); this required one CBCT prior to the first arc, two prior to the second arc, and one prior to the third arc. The mean shifts and rotations following the CBCT alignments immediately before each arc were 0.2 mm and 0.1°. Real-time motion management was performed with Varian OSMS (optical surface monitoring system) throughout the treatment. The average movement detected by OSMS during treatment delivery was <0.2 mm and 0.1° in any direction.

Outcome

This patient was followed clinically and radiographically with contrast-enhanced MRI imaging at three, eight, 14, 20, 25, 28, 32, 35, 38, 45, 60, 72, and 84 months post-SRS treatment. Imaging continues to show a trend toward a reduction in size of the tumor with a nadir that was reached by 60 months post-treatment. Each follow-up MRI was registered in Eclipse with the original planning CT with the new CTV contoured and compared to the original CTV at the time of treatment, as shown in Figure 2. Contouring was performed by a single RO who was doing the follow-ups with the patient. As of 84 months post SRS, the tumor volume was 11% of its original size, indicating that the tumor is at the point of a partial response (>50% reduction in size) [15]. The CTV volume, initially 2.17 cc pre-treatment, was measured to be 0.24 cc in the most recent imaging (84 months).

As of 25 months post-SRS, the patient developed double vision and headaches, and MRI imaging showed the first evidence of enhancement and FLAIR changes indicating the presence of radiation necrosis (RN), which was stable as of the 28- and 32-month post-SRS images and was 3.08 cc in size. Stabilization of these changes after an initial increase is expected based on the presumed diagnosis of necrosis and the expectation that about half of adverse radiation events will improve after 12 months from the initial onset [16]. At the onset of her double vision and the evidence of RN, she was given a tapering prescription of dexamethasone over the course of a month. Between 25 and 32 months post-SRS, the patient noted an increase in headaches and stabilization of the diplopia, and at 28 months post-SRS, she was given another short course of dexamethasone and advised to take vitamin E 400 IU twice daily. Following 28 months post-SRS, the FLAIR enhancement started to resolve fairly rapidly and decreased in size to 0.38 cc by 84 months post-treatment. During this period, she did not receive any further dexamethasone, and she was managed conservatively; headaches and diplopia both improved but remained intermittently present following resolution of the FLAIR changes. Difficulty in distinguishing necrosis exists in many studies, but in this particular case, the enhanced region was outside of the tumor volume and, coupled with the changes in FLAIR imaging, was clearly distinguishable as radio-necrosis.

## Discussion

As pineal parenchymal tumours are uncommon, the most effective treatment approach remains unclear. Treatment options generally depend on the presence of hydrocephalus, surgical resectability, and histology. Treatment can include surgery (resection and/or ventricular drain), chemotherapy, and radiation therapy. When including radiation therapy in the treatment, stereotactic radiosurgery is considered a viable treatment option for PPTs, though large variability in prescriptions has been used with marginal doses ranging from 12 to 20 Gy [6-12]. Traditionally, SRS treatments for PPTs have only been performed using either Gamma Knife or CyberKnife; however, with advances in the accuracy of patient set-up and localization and radiation delivery, traditional linear accelerators are now able to deliver SRS treatments. Outcomes for this patient provide supportive evidence of the effectiveness of SRS, similar to findings of other studies [6-12]; the number of patients presented in the literature is still very limited. This work supplies additional information for possible treatment management.

The delivery accuracy was deemed acceptable and well within the tolerances capable of a linear accelerator, and the patient has had an excellent clinical response. However, given the toxicity with RN, a treatment plan was created for comparison with a 0 mm PTV margin, which would have been used if the patient had been treated with a frame-based rigid immobilization (either on a linac or CK or GK). The plan was optimized to meet the same constraints in Eclipse as the actual treatment plan. With this re-optimized 0 mm margin plan, the brainstem Dmax (0.03cc) was 14.4 Gy, and the healthy brain (brain minus CTV minus CSF) V_10Gy_, V_12Gy_, and V_14Gy_ were 6.3 cc, 3.7 cc, and 2.2 cc, respectively, which are approximately 2-4 cc less than the actual treatment plan. Modeled risks of RN after SRS from the Stereotactic brain HyTEC paper indicate a risk of approximately 11% of symptomatic necrosis for a V_12Gy_ of 6.5 cc. The risk with the 0 mm PTV margin would have decreased to approximately 7% (approximately 4% decrease in risk). From the total irradiated V_14Gy_ of 7.0 cc, there was a 0.4% risk of grade 3 necrosis [17]. The risk of developing RN was low for either a 1 mm or 0 mm margin; however, extra care must still be taken to minimize dose to normal structures outside of the tumor volume. This could have been achieved using either frame-based immobilization, which allows for a 0 mm PTV margin, though considerations need to be made for the increase in resource requirements, as well as being more invasive and inducing more patient discomfort, or by using a lower peripheral dose. The patient will continue with regular follow-up imaging to assess the continued response of the PPT and RN.

## Conclusions

The optimal treatment strategy for PPTs is not currently well known due to their low incidence. Treatment options generally depend on the presence of hydrocephalus, surgical resectability, and histology, and treatment can include surgery (resection and/or ventricular drain), chemotherapy, and radiation therapy. When including radiation therapy in the treatment, stereotactic radiosurgery is considered a viable treatment option for PPTs, although large variability in prescriptions has been used with marginal doses ranging from 12 to 20 Gy. The treatment process developed here, using single-fraction linac-based VMAT SRS for a single patient case, has shown a good response thus far with a marginal prescription dose of 18 Gy. The exact size and location of PPTs and the resulting dosimetric parameters would determine if this approach is suitable for future cases. Risk comparisons with 0 mm margin plans or different prescription doses could be performed to help decide the most appropriate treatment option. Although the patient remains asymptomatic of her original presenting symptoms during ongoing follow-up, the incidence of radio necrosis at 25 months highlights the importance that adverse radiation effects can manifest in a delayed manner, with most cases eventually resolving over time, as seen in this case. Consequently, long-term imaging follow-up remains essential to monitor for potential late effects and to distinguish adverse radiation effects from tumor progression.
